# Association of Glomerular Filtration Rate with Inflammation in Polycystic Ovary Syndrome

**DOI:** 10.22074/ijfs.2015.4238

**Published:** 2015-07-27

**Authors:** Ilay Ozturk Gozukara, Kerem Han Gozukara, Suna Kabil Kucur, Eda Ulku Karakılıc, Havva Keskin, Derya Akdeniz, Ayse Nur Aksoy, Ayse Carlıoglu

**Affiliations:** 1 Department of Obstetric and Gynecology, Dumlupınar University Medical Faculty Hospital , Kütahya, Turkey; 2 Department of Urology, Palandöken Hospital, Erzurum, Turkey; 3 Department of Obstetric and Gynecology, Nenehatun Women Health Hospital, Erzurum, Turkey; 4 Department of Internal Medicine, Erzurum Bölge Training and Research Hospital, Erzurum, Turkey; 5 Department of Internal Medicine, Fatih University Medical Faculty Hospital, Ankara, Turkey

**Keywords:** CRP, Glomerular Filtration Rate, Polycystic Ovary Syndrome, Uric Acid

## Abstract

**Background:**

We aimed to estimate the glomerular filtration rate (GFR) in women with polycystic ovary syndrome (PCOS) and to determine the relationship between GFR with C-reactive protein (CRP) and uric acid.

**Materials and Methods:**

In this cross-sectional study, one-hundred and forty PCOS women and 60 healthy subjects were evaluated. The study was carried out at Endocrinol- ogy Outpatient Clinic, Erzurum Training and Research Hospital, Erzurum, Turkey, from December 2010 to January 2011. GFRs were estimated by Modification of Diet in Renal Disease (MDRD) formula. CRP, urinary albumin excretion (UAE) and uric acid levels were also measured.

**Results:**

GFRs were significantly higher in PCOS group than control (135.24 ± 25.62 vs. 114.92 ± 24.07 ml/min per 1.73 m^2^). CRP levels were significantly higher in PCOS patients (4.4 ± 3.4 vs. 2.12 ± 1.5 mg/l). The PCOS group had significantly higher serum uric acid levels (4.36 ± 1.3 mg/dl vs. 3.2 ± 0.73 mg/dl). There was also significantly higher proteinuria level in PCOS patients.

**Conclusion:**

Even though PCOS patients had higher GFR, serum uric acid and UAE val- ues than control patients, the renal function was within normal limits. Increased GFR in PCOS women positively correlates with elevated serum CRP and uric acid.

## Introduction

Polycystic ovarian syndrome (PCOS) is the most common endocrine disorder affecting 5-10% of women of reproductive age (1). It is characterized by oligo/amenorrhea, hyperandrogenism and polycystic ovaries (2, 3). The insulin resistance, dyslipidemia, glucose intolerance, hypertension and obesity are metabolic disorders accompanying with this syndrome (4-6). It has been assumed that PCOS is also a proinflammatory state. Recent studies have demonstrated that glucose is responsible for inflammatory response in mononuclear cells of women with PCOS independent of body mass index (BMI) (7, 8). There is also an association between inflammation at the molecular level and insulin resistance in this disorder (8, 9). Elevations of a number of circulating proatherogenic inflammatory mediators have been independently reported in PCOS (10, 11). Meta-analysis of the 31 articles reported that circulating C-reactive protein (CRP) was 96% higher in women with PCOS compared to healthy controls (12). The relationship between CRP with atherothrombotic cardiovascular disease and renal function abnormalities has been reported in a number of studies (13).

Serum uric acid was associated positively with interleukin 6 (IL-6), CRP and tumor necrosis factor- alpha (TNF-α) and negatively with IL-1 beta (IL-1β). These results suggest that uric acid contributes to systemic inflammation in humans and is in line with experimental data showing that uric acid triggers sterile inflammation (14). It is also known that hyperuricemia is an independent risk factor for renal dysfunction in the normal population (15).

Urinary albumin excretion (UAE) is also a marker of atherogenesis and predicts early endothelial damage (13). Factors predisposing for endothelial injury, including hyperinsulinemia, insulin resistance, dyslipidemia and chronic low-grade inflammation, which often accompany with PCOS (16). Several studies have shown that microalbuminuria is an indicator for increased permeability to macromolecules of peripheral vascular beds. UAE may predict renal function abnormalities (17).

The aim of this study was to investigate renal function by the way of GFR measurement (MDRD formula) in PCOS patients. We tried to find any relationship between glomerular filtration rate (GFR) with CRP and uric acid as inflammatory markers. Also UAE was evaluated for renal function in PCOS patients.

## Materials and Methods

### Study population

The study was carried out at Endocrinology Outpatient Clinic, Erzurum Training and Research Hospital, Erzurum, Turkey, from December 2010 to January 2011. One-hundred and forty patients with PCOS and 60 healthy subjects were enrolled in this cross-sectional study. We included healthy women as controls with normal menstrual cycles, with no evidence of hyperandrogenism, and with normal ovarian morphology on pelvic ultrasonography. Ferriman- Gallwey scores of all control patients were under 8 (18). PCOS was defined as the presence of two of the following three features after the exclusion of other etiologies (3): i. oligo-or anovulation (fewer than six menstrual periods in the preceding year), ii. hyperandrogenism and/or biochemical signs of hyperandrogenism and/or iii. polycystic ovaries.

All of the participants are nonsmokers and with body mass index (BMI) lower than 25. The exclusion criteria in control and PCOS groups were as follows: patients with any type of renal disease, diabetes mellitus, cardiovascular events, endocrine disease, pregnancy, or antihypertensive drug use including use of oral contraceptives, antidiabetics, glucocorticoids, and anti androgenic agents within the last 3 months. Leukocyte count was less than 10,000/μL in all cases. Patients with older than 40 and younger than 16 years old were excluded from the study.

### Assessments

BMI was calculated as weight (kg)/height (m)^2^. Systolic (SBP) and diastolic blood pressure (DBP) were measured twice in the right arm in a relaxed sitting position. Two measurements were taken 15 minutes apart and the average of two was used. Blood samples were collected during early follicular phase of menstrual cycle after at least 12 hours fasting. Levels of glucose, insulin, serum urea (not blood urea nitrogen), creatinine, hormone profile [follicle-stimulating hormone (FSH), luteinizing hormone (LH), estradiol (E_2_), and thyroidstimulating hormone (TSH)], total and free testosterone (Total-T and Free-T), dehydroepiandrosterone sulfate (DHEAS), 17 OH-progesterone (17OH-P), prolactin (PRL), and serum lipids [total cholesterol (Total-C), high-density cholesterol (HDL-C), lowdensity cholesterol (LDL-C), and triglycerides (TG)] were determined. Plasma glucose was determined with the glucose hexokinase method (Cobas Integra 400 Plus, Roche Diagnostics, Mannheim, Germany). Hormone profile was measured with electrochemiluminescence assays (Elecsys 2010 Hitachi, Roche Diagnostics, Germany). Lipid profile was measured with enzymatic colorimetric assays (Roche Diagnostics, Mannheim, Germany).

Plasma concentrations of insulin were measured by chemiluminescent immunoassay (Immulite One, BioDPC, Los Angeles, CA, USA). Insulin resistance was measured with homeostasis model assessment for insulin resistance (HOMA-IR) (19).

UAE was determined in 24-hour urine samples (Roche/Hitachi 912 Autoanalyzer, Roche Diagnostics, Germany). A UAE of 30-300 mg/24-hour was considered as microalbuminuria, whereas the value >300 mg/24-hour was considered as proteinuria. GFR was estimated from serum creatinine using the MDRD formula (20) as follows:

$$\mathrm{GFR}(\mathrm{ml}/\min/1.73m^{2})=175\times {\mathrm{(Serum Creatinine)}}^{\mathrm{-1.154}}\times {\mathrm{(age)}}^{-0.203}\times 0.742$$

Serum uric acid levels were measured by uricase method using an Abbott Aeroset autoanalyzer (Abbott Laboratories, Abbott Park, IL, USA) with a 0.01 mmol/l limit of detection and mean coefficients of variations <2%.

Serum CRP levels were measured using a nephelometric assay (Boehringer, Mannheim, Germany). Complete blood and polymorphonuclear leukocyte counts (%) were measured with a Coulter MaxM analyzer (Philadelphia, PA, USA).

### Study ethics

The study was conducted according to the revised guidelines for clinical studies described by the World Medical Association’s Declaration of Helsinki (http:// www.wma.net). The study protocol was approved by the Ethical Committee of Erzurum Training and Research Hospital. A written informed consent was obtained from all participants.

### Statistical analysis

All statistical analyses were performed using the Statistical Package for the Social Sciences (SPSS; SPSS Inc., Chicago, IL, USA) version 13.0, while the differences within- or between-group were analyzed by Student’s paired and unpaired t tests. Results were expressed as mean ± standard deviation (SD). Pearson’s correlation was used to calculate correlations. A multiple regression analysis was performed to determine the independent association between potential predictor variables and GFR as the dependent variable. A P value ≤0.05 was considered statistically significant.

## Results

The demographic variables and biochemical features of PCOS and controls women are shown in tables 1 and 2. A hundred and forty PCOS patients (median age: 24.6 ± 5.5 year) and 60 healthy subjects (median age: 25.2 ± 4.38 year, P=0.687) were included in the study. There were no significant differences between groups with respect to age, height, weight, waist circumference, BMI, serum total cholesterol, LDL, HDL, TG, TSH, FSH and E_2_ levels (P>0.05). PCOS group had significantly higher LH values (P=0.02). Both groups were normotensive regarding SBP and DBP (P=0.43, P=0.8, respectively). Serum insulin levels and HOMA-IR were significantly higher in PCOS patients (P=0.02, P=0.007, respectively), while fasting plasma glucose level was not statistically different between two groups (P=0.07). C-reactive protein was significantly higher in PCOS patients (4.4 ± 3.4 vs. 2.12 ± 1.5 mg/l, P=0.01).

The PCOS group had significantly higher serum uric acid (4.36 ± 1.3 vs. 3.2 ± 0.7 mg/dl, P=0.002) beside the fact that statistically similar urea and creatinine levels for each group were reported (P=0.72, P=0.09, respectively). GFR was significantly higher in PCOS group than controls (135.2 ± 25.6 vs. 114.9 ± 24.1 ml/min per 1.73 m_2_, P=0.001).

Multiple regression analysis was performed with GFR as a dependent variable. Some parameters such as glucose, BMI, and HOMA-IR were used as an independent variables. Since obesity and diabetes mellitus can cause hyperfiltration (21, 22), GFR was significantly higher in PCOS group in multiple regression analysis including BMI, HOMA- IR, glucose, age, waist circumference, CRP and insulin.

To assess the correlation with GFR, a Pearson’s correlation analysis was performed on each variable. GFR was positively correlated with uric acid (Correlation of determination=0.065, P=0.01) and CRP (Correlation of determination=0.23, P=0.000).

In PCOS group, UAE ranged from 3 to 105 mg/ml with a median of 13 mg/ml, whereas in control groups, UAE ranged from 2 to 43.8 mg/ml with a median of 7 mg/ml. There was no patient with macroscopic proteinuria in both groups. Mean UAE was statistically higher in PCOS group than controls (P=0.021). Eleven percent of control groups and 28% of PCOS groups had proteinuria. This difference was statistically significant (P=0.02).

**Table 1:** The demographic variables and biochemical parameters of patients


Parameters	PCOS (n=140)	Control (n=60)	P values

Age (Y)	24.6 ± 5.4	25.2 ± 4.38	0.17
Height (m)	1.63 ± 0.07	1.62 ± 0.05	0.68
Weight (kg)	59.6 ± 17.1	55.2 ± 10.4	0.1
BMI (kg/m^2^)	24.6 ± 6.4	22.9 ± 4.3	0.42
Waist circumference (cm)	78.7 ± 15.3	76.7 ± 8.3	0.56
Urea (mg/dL)	23.4 ± 9.6	24.3 ± 7.2	0.72
Crea (mg/dL)	0.83 ± 0.1	0.6 ± 0.12	0.09
GIucose (mg/dl)	89.9 ± 19.7	85.6 ± 6.6	0.07
Insulin (mU/L)	10.4 ± 6.5	7.22 ± 4.33	0.02
HOMA-IR	1.79 ± 1.66	0.85 ± 0.87	0.007
GFR (ml/min per 1.73 m^2^)	135.2 ± 25.6	114.9 ± 24.1	0.001


PCOS; Polycystic ovary syndrome, BMI; Body mass index, Crea; Creatinine, HOMA-IR; Homeostasis model assessment-insuline resistance and GFR; Glomerular filtration rate.

**Table 2:** The biochemical parameters of patients


	PCOS (n=140)	Control (n=60)	P values

Total-C (mg/dL)	166.7 ± 37.3	156.5 ± 23	0.46
LDL-C (mg/dL)	94.2 ± 30	87.3 ± 24.2	0.3
TG (mg/dL)	93.9 ± 52.6	78.8 ± 48.5	0.08
HDL-C (mg/dL)	54.2 ± 17.5	52.6 ± 14.6	0.26
UAE (mg/ ml)	13 ± 6.1	7 ± 3	0.021
Uric acid (mg/dL)	4.36 ± 1.3	3.2 ± 0.7	0.002
CRP (mg/L)	4.4 ± 3.4	2.12 ± 1.5	0.01
TSH (mIU/L)	2.79 ± 1.5	2.91 ± 2.6	0.29
FT3 (pg/dL)	3.1 ± 1.3	3.6 ± 2.3	0.82
FT4 (ng/dL)	3.7 ± 1.8	3.2 ± 1.9	0.73
FSH (mIU/mL)	5.5 ± 1.9	6.5 ± 1.4	0.06
LH (mIU/mL)	8.8 ± 3.5	6.4 ± 4.8	0.02
E2 (pg/mL)	75.6 ± 33	80.8 ± 40.5	0.3
Progesteron (ng/mL)	3.2 ± 2.4	0.73 ± 0.4	0.33
PRL (ng/mL)	10.3 ± 3.5	13.3 ± 3.7	0.2
DHEAS (mcg/dL)	241 ± 96	192 ± 83	0.012
Total-T (ng/dL)	37.6 ± 31	21.7 ± 21	0.001
17 OH-P (ng/mL)	1.2 ± 0.4	1.02 ± 0.04	0.83
Free T (pg/mL)	2.7 ± 0.9	1.7 ± 0.4	0.69
SBP (mmHg)	121.7 ± 13	114.1 ± 11	0.43
DBP (mmHg)	80.8 ± 10.5	74.4 ± 9	0.8


PCOS; Polycystic ovary syndrome, Total-C; Total cholesterol, LDL-C; Low-density cholesterol, TG; Triglycerides, HDL-C; High-density cholesterol, UAE; Urinary albumin excretion, CRP; C- reactive protein, FT3; Free triiodothyronine, FT4; Free thyroxine, FSH; Follicle stimulating hormone, LH; Luteinizing hormone, E_2_; Estradiol, PRL; Prolactine, TSH; Thyroid-stimulating hormone, DHEAS; Dehydroepiandrosterone, Total-T; Total testosterone, 17 OH-P; 17-Hydroxiprogesterone, Free-T; Free testosterone, SBP; Systolic blood pressure and DBP; Diastolic blood pressure.

## Discussion

To our knowledge, this is the first study for the demonstration of significantly higher GFR in PCOS women as compared with the healthy subjects. However, GFR values of PCOS patients were within normal limits. Hyperfiltration is typically defined by a GFR between 125 to 140 ml/min per 1.73 m_2_, or greater than 2 standard deviations above the mean GFR, in healthy individuals (23, 24). According to the National Kidney Foundation (NKF), normal range is defined between 90 and 120 ml/min per 1.73 m_2_ (25). No commonly agreement upon definition of glomerular hyperfiltration exists. Even though in our study, overt hyperfiltration was not found in PCOS patients, they had significantly higher GFR values than controls. Yanes et al. (26) reported increased GFR in a rat model of PCOS. In humans, hyperfiltration is observed in diabetes mellitus patients, and also seen in patients with pre-diabetic conditions, such as the metabolic syndrome (21). Similarly the individuals with obesity exhibit a significant increase in GFR (22). In our study, GFR was also significantly higher in PCOS group in multiple regression analysis including BMI, HOMA-IR, glucose, and insuline. Lakhani et al. (27) has shown that there was no difference in GFR between women with PCOS and controls (102.2 vs. 114.4 ml/min per 1.73 m_2_). However, 15 PCOS patients were included in their study.

There might be vascular and tubular factors contributing to the pathogenesis of hyperfiltration (21). Hyperfiltration is also associated with lower arterial stiffness and endothelial dysfunction, suggesting that hyperfiltration represents a distinct physiologic state of generalized vascular dysfunction. It has, therefore, been suggested that the hyperfiltration state reflects generalized microvascular and macrovascular functional changes (27, 28). In this study, we found relatively higher GFR in PCOS patients.

In the present study, GFR showed a significantly positive correlation with CRP and uric acide. Inflammatory state may be responsible for increased GFR process, which is the result of vascular, tubular and endothelial changes.

CRP is a circulating marker of the proinflammatory state in PCOS as evidenced by the 2-fold elevation in circulating CRP compared to controls (12). Similarly in our study, C-reactive protein was significantly higher in PCOS patients. A metaanalysis of the most comparable studies indicates that elevated circulating CRP in PCOS suggests the chronic low-grade inflammation present in the disorder. They also found that elevated circulating CRP in PCOS is independent of obesity since this finding persisted after excluding all the studies with mismatches in frequency of obesity or BMI between groups from the meta-analysis (12). Although Stuveling et al. (13) showed that elevated CRP was positively associated with diminished filtration, based on our findings, the chronic inflammation in PCOS patients may be responsible for increased GFR levels. On the other hand, in their studies, highest CRP quartile groups were positively associated with hyperfiltration. This association is important because increased GFR is associated with declining renal function (29, 30).

In this study, the PCOS group had significantly higher uric acid, but showed statistically similar urea and creatinine levels with control group. Additionally, increased GFR was positively correlated with uric acid in this paper. It is paradoxical because increased GFR is associated with increased clearance of uric acid from blood that leads to low plasma levels of uric acid. These results may be associated with PCOS itself. Increased uric acid levels in PCOS women were demonstrated in several studies (16).

In the studies, renal dysfunction was correlated with elevated serum uric acid (31-33). Price et al. (34) reported that uric acid is transported into endothelial cells via urate transporter-1, and it then induces oxidative stress. In addition, it has been reported that hyperuricemia increases juxtaglomerular renin expression and decreases macula densa neuronal nitric oxide synthase expression (35). Thus, uric acid may cause renal injury by interacting synergistically with the renin-angiotensin system beside oxidative stress (36). Uric acid has also been shown to directly stimulate the production of inflammatory mediators, such as CRP, in vascular cells (37). In the present study, elevated uric acid levels probably contributed to hyperfiltration like CRP as an inflammatory marker.

In our study, there were significantly higher UAE levels in PCOS group. In one study it appears that excessive UAE may be even more common in PCOS than in subjects with overt diabetes and/or hypertension (38). Urinary excretion of albumin reflects renal function and is directly related to endothelial function or endothelial leakiness. Albumin leakage into the urine is a reflection of widespread vascular dysfunction and increased intraglomerular pressure (39, 40). The National Health and Nutrition Examination Survey (NHANES; 1999-2000) reported the microalbuminuria prevalence as 8.8% in a subpopulation with no risk factors (41). In our study, 11% of control group and 28% of PCOS group showed proteinuria. Ganie et al. (42) have shown that about 24.6% of women with PCOS showed presence of microalbuminuria in the first void spot urine sample.

This study has some limitations. Only CRP and uric acid were used. Mean values of serial measurements of CRP, high-sensitive CRP and other inflammatory markers probably improved study results. Even though relatively higher uric acid, CRP and GFR levels were found in PCOS group, all of these were within normal limits. This may be related with low-grade chronic inflammation (12). Further studies are needed to assess current outcome.

## Conclusion

UAE level and increased GFR are important because they are associated with declining renal function (27). Early inflammatory process may predispose the kidney to glomerular hyperfiltration- related renal function loss. Even though PCOS patients had higher GFR, serum uric acid and UAE levels than control group, they had renal function within normal limits. Further studies may be helpful for understanding PCOS long-term effect on renal function.
